# From guts to skin; unmasking risk factors of pyoderma gangrenosum among Crohn’s disease patients

**DOI:** 10.1007/s00403-024-03119-5

**Published:** 2024-07-05

**Authors:** Camryn Schroeder, Renuka Verma, Lily Liu, Hemamalini Sakhthivel, Kyaw Min Tun, Kamleshun Ramphul, Banreet Singh Dhindsa

**Affiliations:** 1 University of Nevada Las Vegas Kirk Kerkorian School of Medicine, 625 Shadow Lane, Las Vegas, NV 89106 United States; 2 Department of Internal Medicine, University of Nevada Las Vegas, Las Vegas, NV United States; 3 Department of Internal Medicine, Interfaith Medical Center, Brooklyn, NV United States; 4 Independent Researcher, Triolet, Mauritius; 5 NYU Langone Health, , New York, NY United States

Crohn’s disease (CD) is an inflammatory bowel disease with multiple extraintestinal manifestations. Pyoderma gangrenosum (PG), a painful neutrophilic dermatosis, is associated with CD and can require hospitalization [1, 2]. Our goal is to identify comorbidities in CD patients that may influence the incidence of PG.

A retrospective study via the 2001–2020 National(Nationwide) Inpatient Sample, a set of yearly hospital discharge records produced by the Healthcare Cost and Utilization Project (HCUP), was conducted involving patients diagnosed with CD, using pre-tested ICD-9 and ICD-10 codes from previous studies [3]. Our exclusion criteria comprised of patients aged < 18 years and those with a code for Ulcerative Colitis. We used multivariable regression models to investigate multiple comorbidities that may influence the presence of PG. As the NIS consists of de-identified patient samples, the data provider waives the need for IRB approval or institutional ethics clearance. Additional information on the database can be found on: https://hcup-us.ahrq.gov/nisoverview.jsp. SPSS 29.0 (IBM Corp., Armonk, NY) and STATA 18.0(StataCorp. 2023. TX: StataCorp LLC.) were used for our analyses.

In total, we found 3,343,429 cases of CD that matched our selection criteria, with 12,987 adults also having a diagnosis of PG (388 cases of PG per 100,000 hospitalizations of CD). PG patients were younger with a mean age of 45.92 years (vs. 50.76 years, *p* < 0.010), and were more likely to be Females (vs. Males, aOR 1.299, *p* < 0.01), with a history of diabetes (aOR 1.668, *p* < 0.01), obesity (aOR 1.876, *p* < 0.01), and cachexia (aOR 1.534, *p* < 0.01). We also found racial disparities as Blacks (aOR 1.906, *p* < 0.01) and Hispanics (aOR 1.205, *p* < 0.01) with CD were more likely to report PG than Whites.

Furthermore, several comorbid autoimmune conditions such as Sarcoidosis (aOR 2.588, *p* < 0.01), Sjogren disease (aOR 1.534, *p* < 0.01), Rheumatoid Arthritis (aOR 2.068, *p* < 0.01), and atopic dermatitis (aOR 1.892, *p* < 0.01) were common among PG patients. Oral aphthae were also more likely among PG cases (aOR 4.191, *p* < 0.01). However, we found that PG cases were less likely among Medicaid (aOR 0.731, *p* < 0.01), and privately insured patients (aOR 0.608, *p* < 0.01)(vs. Medicare), and also among those with hypertension (aOR 0.783, *p* < 0.01), dyslipidemia (aOR 0.661, *p* < 0.01), smokers (aOR 0.884, *p* < 0.01), or a history of alcohol abuse (aOR 0.604, *p* < 0.010), patients ages ≥ 60 years (vs. 18–59 years, aOR 0.443, *p* < 0.01), with a history of neoplasm (aOR 0.799, *p* < 0.01), or systemic lupus erythematosus (SLE) (aOR 0.703, *p* < 0.01)(Table 1; Fig. 1).

The incidence of PG in CD in our study closely aligns with other published studies [4]. While PG is an uncommon feature among CD patients, our study confirms the potential roles of numerous factors that may influence its presence. The pathophysiology of PG is multifactorial and it is vital to adequately educate CD patients on the signs and symptoms of PG and provide appropriate screening for these patients. Prevention, timely management, and wound care can help reduce additional complications and improve their long-term outcomes [2]. 

**Table 1:** Adjusted odds ratio(aOR), 95% CI, and *p*-values of events of PG among CD cases

Variable	Lower 95% CI	Upper 95% CI	aOR	*p*-value
Females	1.248	1.351	1.299	**< 0.001**
**Medicare as Reference For Primary Payer**				
Medicaid	0.69	0.773	0.731	**< 0.001**
Private insurance	0.579	0.637	0.608	**< 0.001**
**White as Reference for Race**				
Black	1.82	1.996	1.906	**< 0.001**
Hispanic	1.107	1.311	1.205	**< 0.001**
Asian/Pacific Islander	0.981	1.482	1.206	0.076
Hypertension	0.749	0.819	0.783	**< 0.001**
Dyslipidemia	0.62	0.704	0.661	**< 0.001**
Smoking	0.849	0.921	0.884	**< 0.001**
Diabetes	1.584	1.757	1.668	**< 0.001**
Chronic Kidney Disease	0.995	1.149	1.069	0.07
Peripheral Vascular Disease	0.979	1.269	1.115	0.102
Cirrhosis	0.805	1.044	0.916	0.188
Alcohol Abuse	0.527	0.693	0.604	**< 0.001**
Obesity	1.78	1.976	1.876	**< 0.001**
HIV	0.655	1.147	0.866	0.316
Cachexia	1.294	1.818	1.534	**< 0.001**
Ankylosing Spondylitis	0.986	1.537	1.231	0.067
Sarcoidosis	2.129	3.146	2.588	**< 0.001**
Systemic Lupus Erythematosus	0.59	0.837	0.703	**< 0.001**
Celiac	0.504	1.009	0.713	0.057
Sjogren	1.154	2.039	1.534	**0.003**
Rheumatoid Arthritis	1.919	2.23	2.068	**< 0.001**
Oral Aphthae	3.268	5.375	4.191	**< 0.001**
Hyperthyroidism	0.601	1.081	0.806	0.15
Hypothyroidism	0.873	1.071	0.967	0.517
Atopic Dermatitis	1.472	2.433	1.892	**< 0.001**
Neoplasm	0.747	0.854	0.799	**< 0.001**
Age 60 and more	0.42	0.468	0.443	**< 0.001**
Charlson Comorbidity Index (CCI) Score ≥ 3	0.898	1.015	0.955	0.141

**Fig. 1:**
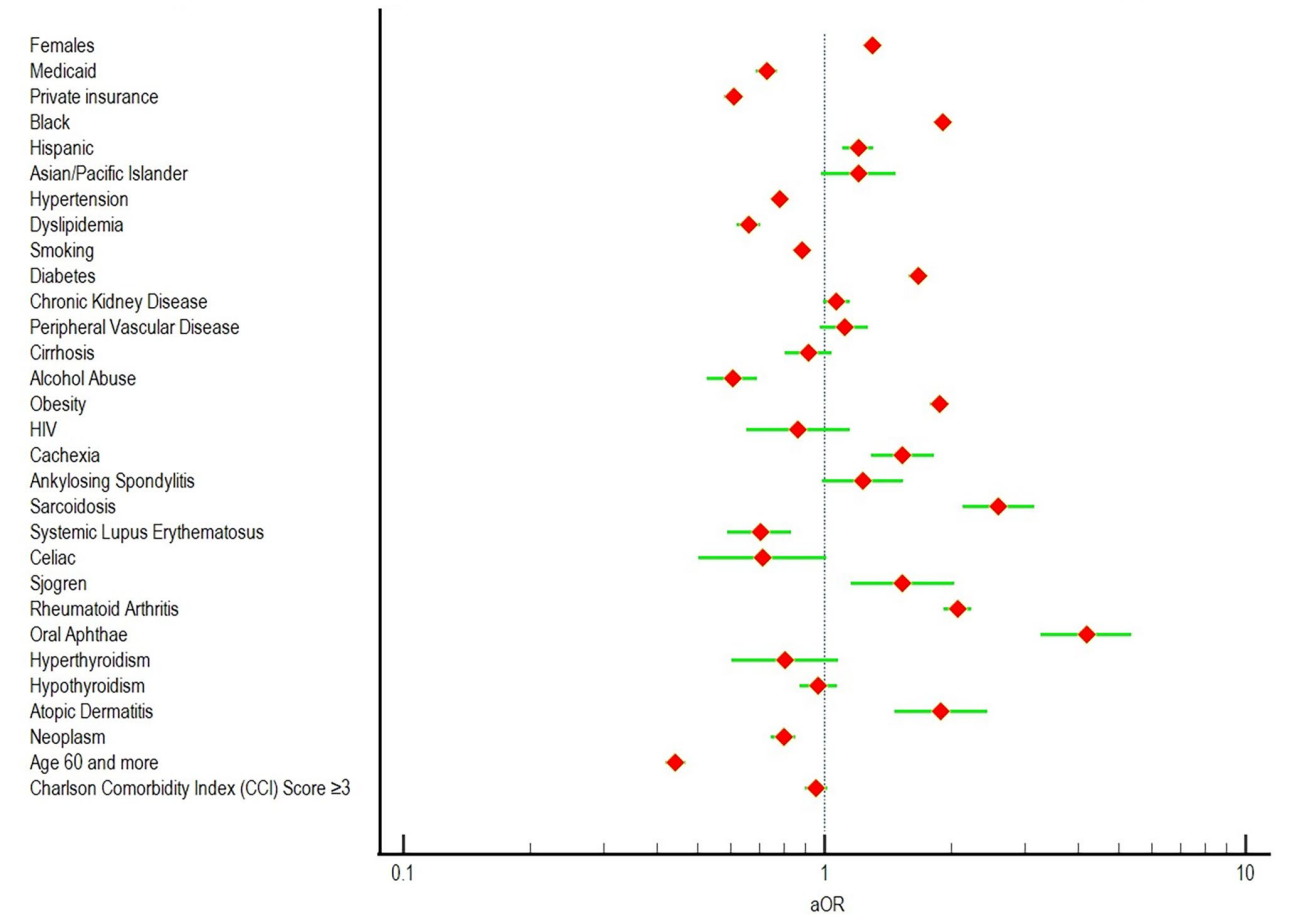
Forest plot of aOR of events of PG among CD cases

## Data Availability

No datasets were generated or analysed during the current study.
